# Food insufficiency and mental health service utilisation in the USA during the COVID-19 pandemic

**DOI:** 10.1017/S1368980021003001

**Published:** 2021-07-15

**Authors:** Jason M Nagata, Kyle T Ganson, Chloe J Cattle, Henry J Whittle, Alexander C Tsai, Sheri D Weiser

**Affiliations:** 1 Department of Pediatrics, University of California, San Francisco, 550 16th Street, 4th Floor, Box 0110, San Francisco, CA94158, USA; 2 Factor-Inwentash Faculty of Social Work, University of Toronto, Toronto, ON, Canada; 3 Division of Psychiatry, University College London, London, UK; 4 Center for Global Health, Mongan Institute, Massachusetts General Hospital; Harvard Medical School, Boston, MA, USA; 5 Division of HIV, Infectious Diseases and Global Medicine, School of Medicine, University of California, San Francisco, San Francisco, CA, USA

**Keywords:** Food insufficiency, Food insecurity, Severe acute respiratory syndrome coronavirus 2, SARS-CoV-2, COVID-19, Mental health

## Abstract

**Objective:**

To estimate the association between food insufficiency and mental health service utilisation in the USA during the COVID-19 pandemic.

**Design:**

Cross-sectional study. Multiple logistic regression models were used to estimate the associations between food insufficiency and mental health service utilisation.

**Setting:**

US Census Household Pulse Survey data collected in October 2020.

**Participants:**

Nationally representative sample of 68 611 US adults.

**Results:**

After adjusting for sociodemographic factors, experiencing food insufficiency was associated with higher odds of unmet mental health need (adjusted OR (AOR) 2·90; 95 % CI 2·46, 3·43), receiving mental health counselling or therapy (AOR 1·51; 95 % CI 1·24, 1·83) and psychotropic medication use (AOR 1·56; 95 % CI 1·35, 1·80). Anxiety and depression symptoms mediated most of the association between food insufficiency and unmet mental health need but not the associations between food insufficiency and either receiving mental health counselling/therapy or psychotropic medication use.

**Conclusions:**

Clinicians should regularly screen patients for food insufficiency, especially in the wake of the COVID-19 pandemic. Expanding access to supplemental food programmes may help to mitigate the need for higher mental health service utilisation during the COVID-19 pandemic.

Food insecurity exists when households struggle to provide adequate and consistent nutrition for one or more members due to the lack of access, money or other resources^(1)^. Food insufficiency, a related, but more narrowly defined concept that has been used by federal surveys for decades, is characterised by not having enough food to eat (i.e. over a specified time period)^(2)^. Over the past 5 years, the United States Department of Agriculture has estimated that the prevalence of food insecurity has steadily ranged from 11 to 12 % of households^(1)^. Since the onset of the COVID-19 pandemic, however, food insecurity in the USA has doubled overall and tripled in households with children^(3)^. Despite measures by Congress to temporarily expand federal food assistance programmes, food insecurity and food insufficiency remain persistently elevated^(4)^.

Food insecurity has been associated with wide-ranging deleterious health outcomes, especially substance use and mental health disorders, including depression, anxiety and sleep disorders^(5–11)^. Studies have reported a bidirectional relationship between food insecurity and negative emotional well-being^(7)^, as having consistent access to nutritious food is known to be important for maintaining physical and mental well-being. Additionally, those who are affected by poor mental health are more likely to have difficulty getting the time, money and energy necessary to provide food for themselves and/or their households and may struggle to address these competing demands^(7)^. The COVID-19 pandemic has ushered in a mental health crisis of its own, with concerning rises in symptoms of depression, anxiety and PTSD observed across countries and age groups^(12–14)^. A recent study during the pandemic found that food insufficiency was associated with symptoms of depression and anxiety^(15)^. The present study builds directly on this previously published work to estimate the associations between food insufficiency and three different measures of mental health care utilisation, using the same nationally representative sample.

Although food insecurity is an independent risk factor for mental illness, those who experience food insecurity may also have difficulty obtaining adequate and consistent mental health care, contributing to even greater mental health morbidity. In a study of adults with severe mental illness in the USA, those with food insecurity were more likely to report less mental health service utilisation^(16)^. On the other hand, food insecurity has been associated with increased medical and psychiatric emergency service utilisation^(17–21)^. In a study of women with HIV, moderate food insecurity was associated with increased use of psychotropic medications, while severe food insecurity was associated with decreased use^(22)^. Collectively, these findings suggest that those with food insecurity have elevated mental health needs but may face increased barriers to accessing these services. As a result, they may engage less with primary care services but may have increased acute care utilisation^(23)^.

This complex relationship has yet to be explored in the context of the COVID-19 pandemic, during which new barriers to health care access are likely to have arisen. As the pandemic continues and food insecurity and mental health become increasingly important national health concerns, there is a pressing need to understand how food insufficiency affects engagement with mental health services. The purpose of this study was thus to estimate the association between food insufficiency and mental health service utilisation, and the extent to which mental health symptoms mediate the relationship, using weekly national Census data in the USA during the COVID-19 pandemic.

## Methods

In the present study, we analysed data from the weekly, cross-sectional Household Pulse Survey (*n* 68 611) from 14 to 26 October 2020. Conducted by the US Census Bureau in collaboration with multiple federal agencies, the Household Pulse Survey was developed to assess the social and economic impacts of the COVID-19 pandemic. The Household Pulse Survey utilised the Census Bureau’s Master Address Files to generate the sample, and selected households were contacted via text messaging and/or email. The Qualtrics online platform was used to collect the de-identified data, which is now publicly available on the Census Bureau website, along with additional details about the Household Pulse Survey study and data collection process^(24)^. To address non-response bias, sample weights were applied based on the demographic distribution of the respondents. Missing data for each variable are shown in Appendix A; a complete case analysis was conducted.

### Measures

#### Dependent variables

Mental health care service utilisation was assessed by using three different measures, each corresponding to a unique binary (yes/no) questionnaire item. Use of prescription psychotropic medications was assessed with the following question: ‘At any time in the last 4 weeks, did you take prescription medication to help you with any emotions or with your concentration, behavior, or mental health?’ Engagement in therapy was assessed by asking participants: ‘At any time in the last 4 weeks, did you receive counseling or therapy from a mental health professional such as a psychiatrist, psychologist, psychiatric nurse, or clinical social worker?’ Finally, the presence of an unmet mental health need was determined by a response of ‘yes’ to the following questionnaire item: ‘At any time in the last 4 weeks, did you need counseling or therapy from a mental health professional, but DID NOT GET IT for any reason?’

#### Independent variables

Participants were asked, ‘In the last 7 d, which of these statements best describes the food eaten in your household?’ Response options included ‘Enough of the kinds of food (I/we) wanted to eat’, ‘Enough, but not always the kinds of food (I/we) wanted to eat’, ‘Sometimes not enough to eat’ and ‘Often not enough to eat’. The former two response options were coded as food sufficient, while the latter two were coded as food insufficient, consistent with the US Household Food Security Survey Module^(25)^.

#### Mediator variable

Depressive symptoms were assessed using an adapted version of the Patient Health Questionnaire for Depression and Anxiety^(26)^.

### Statistical analysis

Unadjusted differences were calculated using independent samples *t* tests (continuous variables) or Pearson’s *χ* ^2^ tests (categorical variables). Multiple logistic regression analyses were used to estimate the associations between food insufficiency and mental health service utilisation. Model 1 is unadjusted, and model 2 adjusts for sociodemographic covariates. Sociodemographic covariates were chosen based on previous theory and literature as being potential confounders for the association between food insecurity and mental health service utilisation^(15,16,22)^. We also examined the extent to which depression and anxiety symptoms mediated the association between food insecurity and mental health service utilisation^(27)^. Nonresponse sample weighing was applied to all analyses. Analyses were conducted using Stata 15.1 (StataCorp.) in 2021.

## Results

Demographic characteristics of the participants included in the sample, presented by food sufficiency status, are shown in Table 1. Overall, 11 % of participants reported food insufficiency in the past week. Unmet mental health need, receiving mental health counselling and psychotropic medication use were higher among people with food insufficiency compared with those without food insufficiency.

**Table 1:** Demographic characteristics of US Census Household Pulse Survey by food sufficiency status, October 2020 (*n* 68 611)

	Total	Food sufficient	Food insufficient	*P*
	%*	%*	%*	
Demographics				
Age (years)				
Mean	48·03	49·35	43·87	<0·001†
se	0·15	0·16	0·48	
Sex				0·020‡
Female	51·67 %	51·66 %	55·20 %	
Male	48·33 %	48·34 %	44·80 %	
Race/ethnicity				<0·001‡
White alone, not Hispanic	62·71 %	66·49 %	46·26 %	
Black alone, not Hispanic	11·52 %	9·84 %	19·25 %	
Asian alone, not Hispanic	4·99 %	5·19 %	2·55 %	
Two or more races + Other races, not Hispanic	3·89 %	3·65 %	5·89 %	
Hispanic or Latino (may be of any race)	16·89 %	14·82 %	25·94 %	
Married	55·42 %	58·87 %	37·03 %	<0·001‡
Number of children in household				
Mean	0·75	0·69	1·09	<0·001†
se	0·01	0·01	0·04	
Number of adults in household				
Mean	2·63	2·58	2·81	<0·001†
se	0·01	0·02	0·05	
Below federal poverty level	12·50 %	11·26 %	37·88 %	<0·001‡
Education				<0·001‡
High school or less	39·07 %	34·97 %	58·62 %	
More than high school	60·93 %	65·03 %	41·38 %	
Food insufficiency, past 7 d	10·86 %	–	–	
Unmet mental health need	10·56 %	8·98 %	23·99 %	<0·001‡
Received mental health counselling	10·01 %	9·42 %	14·94 %	<0·001‡
Psychotropic medication	20·26 %	19·42 %	27·41 %	<0·001‡
Anxiety and depression symptoms (PHQ-4)				
Mean	7·82	7·45	10·98	<0·001†
se	0·03	0·03	0·12	

PHQ-4, Patient Health Questionnaire for Depression and Anxiety.

* All means and percentages are calculated with weighted data to reflect the representative proportion in the target US population.

† Independent samples *t* tests.

‡ Pearson’s *χ* ^2^ tests.

Table 2 shows unadjusted (model 1) and adjusted (model 2) associations between food insufficiency and mental health service utilisation. In models adjusting for sociodemographic factors, experiencing food insufficiency was associated with an unmet mental health need (adjusted OR 2·90; 95 % CI 2·46, 3·43), receiving mental health counselling or therapy (adjusted OR 1·51; 95 % CI 1·24, 1·83) and psychotropic medication use (adjusted OR 1·56; 95 % CI 1·35, 1·80).

**Table 2:** Associations between food insufficiency and mental health service utilisation in US census household pulse survey, October 2020*

Mental health services, past 4 weeks	Unmet mental health need			Received mental health counselling			Psychotropic medication		
	OR	95 % CI	*P*	OR	95 % CI	*P*	OR	95 % CI	*P*
Model 1: Unadjusted									
Food insufficiency, past 7 d	3·11	2·68, 3·61	<0·001	1·62	1·35, 1·94	<0·001	1·51	1·33, 1·72	<0·001
	AOR	95 % CI	*P*	AOR	95 % CI	*P*	AOR	95 % CI	*P*
Model 2: Adjusted									
Food insufficiency, past 7 d	2·90	2·46, 3·43	<0·001	1·51	1·24, 1·83	<0·001	1·56	1·35, 1·80	<0·001

AOR, adjusted OR; SNAP, Supplemental Nutrition Assistance Program.

* The estimated OR in the cells represent abbreviated output from a series of logistic regression models specifying the column header as the dependent variable and the row header as the primary explanatory variable of interest. Thus, the table represents the output from six regression models in total. Adjusted estimates represent abbreviated output from logistic regression models including covariate adjustment for age, sex, race/ethnicity, marriage status, number of children in household, number of adults in household, income and education.

We find that anxiety and depression symptoms mediate 76·1 % (95 % CI 64·7, 92·1) of the association between food insufficiency and unmet mental health need. Anxiety and depression did not appear to mediate the association between food insufficiency and receiving mental health counselling/therapy or between food insufficiency and psychotropic medication use; the estimated regression coefficient for food insufficiency reverses direction and is <1 (and is statistically significant in the case of the model for psychotropic medication) when anxiety and depression are added to the models.

## Discussion

In this nationally representative sample collected during the COVID-19 pandemic, we found that food insufficiency was associated with over a threefold higher odds of unmet mental health need. Food insufficiency was also associated with receiving mental health counselling and higher psychotropic medication use.

Food insecurity/insufficiency has consistently been associated with poor mental health in previous studies, including depressive symptoms, anxiety and suicidality^(7,8,10,15,16,28)^. This relationship explained much of the observed association between food insufficiency and unmet mental health need, operationalised as the inability to access professional mental healthcare in the last 4 weeks despite needing to do so. In addition to the role played by symptoms of poor mental health, the experience of food insufficiency may independently present barriers to accessing ambulatory outpatient mental health services^(16,22)^. The experience of food insufficiency could lead affected people to prioritise food over other needs, using up considerable time and energy to navigate food pantries, free meal services, public sector bureaucracies and other institutions that comprise the US social safety net, or locate and visit affordable food stores. Scarce financial resources are often used to obtain food and are, therefore, not available to fund transport and healthcare charges^(29,30)^. Food insufficiency also depletes energy, lowers mood, and increases anxiety, which have been found to decrease the capacity of individuals to attend healthcare appointments^(23)^.

Conversely, previous studies have also suggested that food insecurity may increase the odds of crisis presentation to emergency psychiatric services^(31)^. This is the same pattern that is seen in studies of food insecurity and general medical care, where most research has focused on HIV: medium- to long-term healthcare is poorly accessed among food-insecure individuals, leading to chronically deteriorating health and, hence, more frequent acute presentations^(32,33)^. In the context of mental health, this pattern of healthcare utilisation may ultimately increase the likelihood of food-insecure people taking psychotropic medications or undergoing counselling, despite poor access to services – such individuals’ mental health tends to deteriorate more severely and they, therefore, eventually come to the attention of providers.

The pandemic has ushered in a financial crisis, associated with unprecedented levels of food insecurity, as well as a mental health crisis. Our findings emphasise the connection between the two and suggest the importance of addressing mental illness as a biopsychosocial phenomenon, with both social-structural interventions (e.g. supplemental food programmes) and medical interventions (e.g. improving access to mental health care services). The persistence of an association between food insecurity and unmet mental health need, despite associations of food insecurity with psychotropic medication use and mental health counselling, suggests that the mental health consequences of food insecurity are not being adequately addressed.

### Limitations

There are a few limitations to this study. The cross-sectional nature of this study limits the ability to infer causality from the observed association between food insufficiency and mental health care utilisation. All measures used in the study are self-reported and are therefore susceptible to recall and reporting bias, but these effects may be attenuated by the anonymity of the online survey. The Census Household Pulse Survey used a single-item measure that was then extrapolated to a binary measure of food insufficiency. While this is an important limitation, many previously published studies also used a similar single-item measure^(34–38)^ and prior research has demonstrated the ability to estimate food insecurity using food insufficiency measures from the Census Household Pulse Survey^(3)^. Similarly, the dependent variables are binary; therefore, we cannot assess frequency of mental health utilisation among the participants included in the sample.

### Clinical and policy implications

The COVID-19 pandemic has taken an alarming toll on mental health and food insecurity in the USA^(3,39)^ and has exacerbated pre-existing racial and ethnic disparities in health^(40,41)^. Already among the nation’s top public health concerns prior to the pandemic, food insecurity and mental illness must be addressed urgently by policymakers and providers. Based on our findings that food insufficiency is associated with increased mental health care utilisation and unmet behavioural health needs, expanding access to supplemental food programmes should be central to pandemic relief efforts. Providers should continue to routinely screen for food insecurity, as recommended by multiple medical associations, and offer referrals to federal and non-governmental food aid programmes^(42)^. The American Rescue Plan Act of 2021 provides $12 billion of funding for food assistance programmes such as SNAP and the Special Supplemental Nutrition Program for Women, Infants, and Children. Specifically, the 15 % increase in SNAP benefits has been extended through September 2021. Still, the sequelae of the pandemic will undoubtedly continue to impact Americans, and more sustainable solutions are needed. Policymakers should support long-term expansion of funding to these programs, with a particular focus on supporting vulnerable communities that have been disproportionately affected.

## Supplementary material

For supplementary material accompanying this paper visit http://dx.doi.org/10.1017/S1368980021003001. click here to view supplementary material
