# Supplementary material for: Food insufficiency and mental health service utilisation in the USA during the COVID-19 pandemic
Source: Public Health Nutr. 2021 Jul 15;25(1):76–81. doi: 10.1017/S1368980021003001 (PMC8367866; doi:10.1017/S1368980021003001)
Supplement: Supplementary file 1 [file S1368980021003001sup001.docx]

| Appendix A. Missing data for each variable in the analysis | | |
| --- | --- | --- |
|  | Missing | Percent missing |
| N=88,716 |  |  |
| Age (per 10 years) | 0 | 0.0% |
| Sex | 0 | 0.0% |
| Race/ethnicity | 0 | 0.0% |
| Number of children in household | 0 | 0.0% |
| Number of adults in household | 0 | 0.0% |
| Married | 754 | 0.9% |
| Income below federal poverty line | 19321 | 21.8% |
| Education (high school or less) | 0 | 0.0% |
| Food insufficiency | 7845 | 8.8% |
| Unmet mental health need | 14611 | 16.5% |
| Received mental health counseling | 14651 | 16.5% |
| Psychotropic medication | 14619 | 16.5% |
| PHQ4 | 13529 | 15.3% |
